# The Expression of P35 Plays a Key Role in the Difference in Apoptosis Induced by AcMNPV Infection in Different *Spodoptera exigua* Cell Lines

**DOI:** 10.3390/ijms241713228

**Published:** 2023-08-25

**Authors:** Qianlong Yu, Minghui Wang, Xuemeng Ding, Jiachen Han, Hancheng Ma, Jie Li, Guiling Zheng, Bin Zhang, Changyou Li

**Affiliations:** Shangdong Engineering Research Center for Environment-Friendly Agricultural Pest Management, Qingdao Agricultural University, Qingdao 266109, China; qlyu@qau.edu.cn (Q.Y.); wmh11j@163.com (M.W.); dingxm98@126.com (X.D.); hanjiachen1219@163.com (J.H.); 15666821220@163.com (H.M.); lijiepd@163.com (J.L.); glzheng@qau.edu.cn (G.Z.); binzhang@qau.edu.cn (B.Z.)

**Keywords:** AcMNPV, apoptosis, *caspase gene*, P35, *Spodoptera exigua*

## Abstract

Baculovirus infection induces apoptosis in host cells, and apoptosis significantly affects virus production. *Autographa californica multiple nucleopolyhedrovirus* (AcMNPV) can regulate apoptosis, but the regulatory mechanism is unclear. Here, we found that AcMNPV infection induced different apoptosis responses in different *Spodoptera exigua* cell lines. In the early stages of viral infection (1–6 h), Se-1 cells underwent severe apoptosis, while Se-3 cells underwent very slight apoptosis. In the late stages of viral infection (12–72 h), Se-1 cells continued to undergo apoptosis and formed a large number of apoptotic bodies, while the apoptosis of Se-3 cells was inhibited and no apoptotic bodies were formed. To determine the reasons for the apoptosis differences in the two cell lines, we measured the expression of the six *S. exigua* cysteine-dependent aspartate specific protease genes (*SeCaspase-1* to *-6*) and the three AcMNPV antiapoptotic protein genes (*iap1*, *iap2* and *p35*) during viral infection. We found that *SeCaspase-1 to -6* were all activated in Se-1 cells and inhibited in Se-3 cells, whereas *iap1*, *iap2* and *p35* were all inhibited in Se-1 cells and normally expressed in Se-3 cells. And *p35* was expressed earlier than *iap1* and *iap2* in Se-3 cells. Otherwise, Se-1 and Se-3 cells would all be apoptotic when infected with the recombinant *p35* knockout AcMNPV, whereas only Se-1 cells were apoptotic, but Se-3 cells were not apoptotic when infected with the recombinant *p35* repair AcMNPV. Combined with the fact that the expression of P35 protein is inhibited in Se-1 cells but normally expressed in Se-3 cells during the infection of recombinant *p35* repair AcMNPV, we proposed that the different expression of P35 is an important reason for the apoptosis differences between the two cell lines. We also found that some genes associated with apoptosis can probably regulate the expression of P35. However, the major upstream regulators of P35 and their mechanisms are still unclear and will be studied in the future.

## 1. Introduction

Baculoviruses are insect-specific viruses with an envelope and double-stranded DNA. These viruses are isolated from the infected insects of Lepidoptera, Diptera, and Hymenoptera and have been developed as insecticides that are widely used for the biological control of insect pests [1,2]. *Autographa californica multiple nucleopolyhedrovirus* (AcMNPV) is a representative species of baculovirus and plays an important role in the biological control of lepidopteran pests such as *Trichoplusis ni*, *S. exigua* and *S. littoralis* [3,4,5]. In an infection cycle, AcMNPV replicates in the nucleus and produces two kinds of virions with distinct phenotypes: occlusion-derived virions (ODVs) and budded virions (BVs). ODVs infect midgut epithelial cells and are included by polyhedrin in the cell nucleus to form occlusion bodies (OBs). BVs infect cells from other tissues and cells cultured in vitro [1,2]. However, BV infection usually triggers cell apoptosis, which affects viral replication, dissemination and infectivity [6]. Interestingly, AcMNPV can encode antiapoptotic proteins to inhibit apoptosis and facilitate their own proliferation and transmission [7,8,9].

Apoptosis is one of the most important insect-immune system responses, resulting in reduced viral replication, dissemination and infectivity [6]. Cysteine-dependent aspartate-specific proteases (caspases) are the core genes that regulate the process of apoptosis [10]. Based on their different positions and functions in the process of apoptosis, the caspase family is divided into initiators and effectors [11]. Initiator caspases are self-activating in the apoptotic response triggered by a virus or other pathogen. They activate downstream effector caspases, which can degrade the cytoskeleton and other related substrate proteins that cause the cell to breakdown into apoptotic bodies [12]. To date, sixteen and seven caspases have been described in mammals (Caspase-1 to -16) and Drosophila (Dronc, Dredd, Drice, Dcp-1, Decay, Dammn and Dream/Strica), respectively [13,14]. Among them, Caspase-2, -8, -9 and -10 of humans and Dronc and Dredd of Drosophila have been shown to be initiator caspases, and Caspase-3, -6 and -7 of humans and Drice and Decay have been indicated to be effector caspases [13,14]. Interestingly, several human initiator caspases, such as Caspase-8 and Caspase-9, and effector caspases, such as Caspase-3 and Caspase-7, have been demonstrated to be involved in cell apoptosis induced by viral infection, and inhibition of their activity can prevent virus-induced apoptosis [15,16,17,18]. In lepidopteran species, six caspases (LepCaspase-1 to -6) have been discovered [19,20]. LepCaspase-1, -2, -3 and -4 are homologous to Drice, Dcp-1, Decay and Dream/Strica, respectively. Among them, LepCaspase-1 has been reported to be an effector caspase [19,21]. LepCaspase-5 and -6 are homologous to Dronc and Dredd, respectively, and they are also homologous to human Caspase-9 and Caspase-8 [19]. LepCaspase-5 has been reported to be an initiator of caspase [22].

Baculoviruses encode four antiapoptotic proteins: inhibitors of apoptosis proteins (IAPs), P35, P49 and apoptosis suppressor (Apsup) [23,24]. The mechanism of these antiapoptotic proteins is similar in that they all inhibit the activity of caspase proteins to inhibit apoptosis [25]. P35 inhibits the activities of both initiator and effector caspases by directly interacting with them [26,27,28]. P49 and Apsup are homologues of P35, and their mechanisms of apoptosis inhibition are similar to those of P35 [29,30,31]. IAPs have been found in nearly all baculoviruses and are classified into six groups, but only a few of them can inhibit apoptosis by interacting with initiator caspases or hijacking host cell IAPs to prevent initiator caspase activation [32,33,34]. AcMNPV encodes three antiapoptotic proteins: IAP1, IAP2 and P35. Perhaps P35 plays a major role in inhibiting AcMNPV-induced apoptosis because IAP1 and IAP2 were unable to inhibit apoptosis in a variety of cell lines during virus infection [35,36].

In this study, we found that AcMNPV infection induced different apoptotic phenomena in the two different *S. exigua* cell lines: during virus infection, Se-1 cells underwent severe apoptosis and apoptosis significantly reduced virus production. However, Se-3 cells underwent slight apoptosis, which was probably suppressed by the virus. By sequence alignment with other lepidopteran caspases [19], we found six caspase genes in the transcriptome of *S. exigua* (*SeCaspase-1* to *-6*). Based on the different roles of caspases and viral antiapoptotic proteins in apoptosis [19,35,36], we speculated that *SeCaspase-1* to *-6* and the antiapoptotic genes AcMNPV, *iap1*, *iap2* and *p35* may be differentially expressed between Se-1 and Se-3 cell lines, and the expression of P35 may play a major role in the apoptosis differences between the two cell lines. To confirm this hypothesis, we determined the expression of these genes during AcMNPV infection. To clarify the important role of *p35*, we infected the two cell lines with recombinant AcMNPV (with *p35* knockout and repair) and detected whether the P35 protein was expressed during viral infection. We also analysed the relationship between the expression of *SeCaspases* and *p35*. Furthermore, we analysed the differentially expressed genes (DEGs) involved in some apoptosis-associated pathways in Se-1 and Se-3 cell lines based on the transcriptome of the two cell lines to explore the upstream regulators of P35.

## 2. Results

### 2.1. Apoptosis and Virus Production Analysis of the Two Different S. exigua Cell Lines Infected with AcMNPV

**Figure 1:**
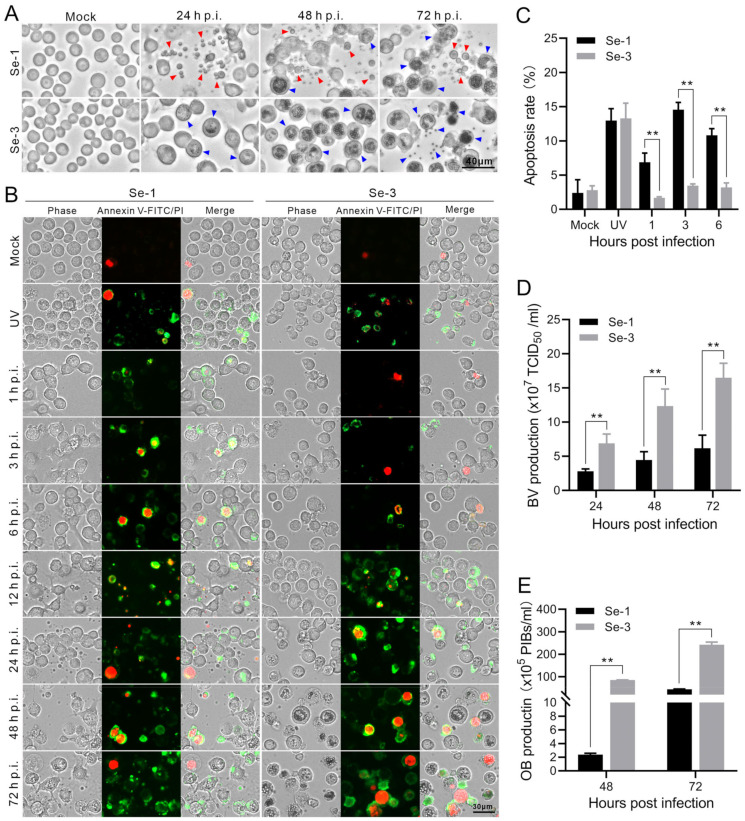
Apoptosis and virus production analysis of the two different *S. exigua* cell lines infected with AcMNPV. (**A**) Apoptosis analysis of Se-1 and Se-3 cell lines. Se-1 and Se-3 cells were infected with wild-type AcMNPV at a multiplicity of infection (MOI) of 5. At 24, 48 and 72 h post-infection (h p.i.), the two infected cell lines were photographed under a microscope. The red arrow indicates the apoptotic bodies, and the blue arrow indicates the OBs. (**B**) Analysis of the apoptosis process of Se-1 and Se-3 cell lines. Se-1 and Se-3 cells were infected with AcMNPV at an MOI of 5. At 1, 3, 6, 12, 24, 48 and 72 h p.i., the apoptosis of the two infected cells was subjected to analysis by Annexin V-FITC/PI and then observed and photographed under a fluorescence microscope. The membranes of apoptotic cells were labelled with Annexin V-FITC in green, and the nuclei of apoptotic cells were labelled with PI in red. Mock cells were the negative control, with no virus infection. UV was a positive control, with the cells exposed to ultraviolet radiation for 4 h. (**C**) The apoptosis rate of Se-1 and Se-3 cell lines. The total number of cells stained green and red was used as the number of apoptotic cells, and the percentage of the total number of apoptotic cells to the total number of cells was used as the apoptosis rate. (**D**) The production of BVs. BV titers were determined by the TCID_50_ assay. (**E**) The production of OBs. The number of OBs produced in the cells was calculated. Each test was performed three times. The error bars indicate the standard error of the mean (SEM) from the means of results for means of triplicates. **, *p* < 0.001.

To explore whether AcMNPV infection induces apoptosis in *S. exigua*, we infected two different *S. exigua* cell lines, Se-1 and Se-3, with AcMNPV at MOIs of 5 and 0.5. At 24, 48 and 72 h post-infection (h p.i.), we investigated apoptosis in the two infected cell lines. The results showed that the Se-1 cells underwent apoptosis, producing apoptotic bodies at 24 h to 72 h p.i. In contrast, the Se-3 cells seemingly did not undergo apoptosis, with no apoptotic bodies produced at any time during AcMNPV infection at an MOI of five (Figure 1A). The apoptosis responses of the two cell lines at an MOI of 0.5 were similar to those at an MOI of five, which is not displayed. In addition, to determine whether apoptosis affects AcMNPV production, infectious BV titers were detected by half-tissue culture infection dose (TCID_50_) assays. OB production was calculated using a hemocytometer at 24 h to 72 h p.i. We found that the BV titer and OB production of Se-1 cells were lower than those of Se-3 cells (Figure 1D,E). Moreover, we also observed that the time of OB generation in Se-1 cells was later than that in Se-3 cells (Figure 1A). It is expected that the apoptosis of Se-1 cells affected the production of both virions.

### 2.2. Analysis of the Apoptosis Process in Two Different S. exigua Cell Lines Infected with AcMNPV

To analyse the apoptosis differences in the infected Se-1 and Se-3 cell lines, we used annexin V-conjugated FITC/PI to identify the differences in the apoptosis processes of the two infected cell lines. The mock cells that were not infected with the virus served as a negative control, and the cells exposed to ultraviolet (UV) radiation for 4 h served as a positive control. The cell membranes of early apoptotic cells were stained green by annexin V-FITC, and the nuclei of late apoptotic cells were stained red by PI. Otherwise, the apoptosis rate was calculated to measure the degree of apoptosis. We found that a few mock cells of Se-1 and Se-3 cell lines had their nuclei stained red, and the apoptosis rates of the two cells were almost the same, both of which were approximately 3% (Figure 1B,C). In contrast, after treatment with UV, many Se-1 and Se-3 cells underwent apoptosis, with their membranes stained green and their nuclei stained red, and the apoptosis rates of the two cell lines were also the same, as both were approximately 13% (Figure 1B,C). Interestingly, infected with AcMNPV, at 1 to 6 h p.i., many Se-1 cells underwent apoptosis with their membranes stained green, and only a few cells had their nuclei stained red. The apoptosis rate of Se-1 cells was approximately 7–14%, whereas a few Se-3 cells also underwent apoptosis with their membranes stained green and nuclei stained red. The apoptosis rate of Se-3 cells was only approximately 2–4% (Figure 1B,C). However, at 12 to 72 h p.i., we could not accurately calculate the apoptosis rate because the number of apoptotic Se-1 cells (with their membranes stained green) increased and these cells degraded into apoptotic bodies (Figure 1B, Se-1 panel). Some Se-3 cells were also stained green and red, but they were not degraded to apoptotic bodies (Figure 1B, Se-3 panel). These results showed that Se-1 cells remained apoptotic during the entire viral infection, while Se-3 cells underwent slight apoptosis in the early stages of infection, but it was probably suppressed in the late stages of infection. This suggested that there was a difference in apoptosis in Se-1 and Se-3 cell lines induced by AcMNPV infection.

### 2.3. Transcription Analysis of the Core Apoptosis Gene Caspases in Two Different S. exigua Cell Lines during AcMNPV Infection

**Figure 2:**
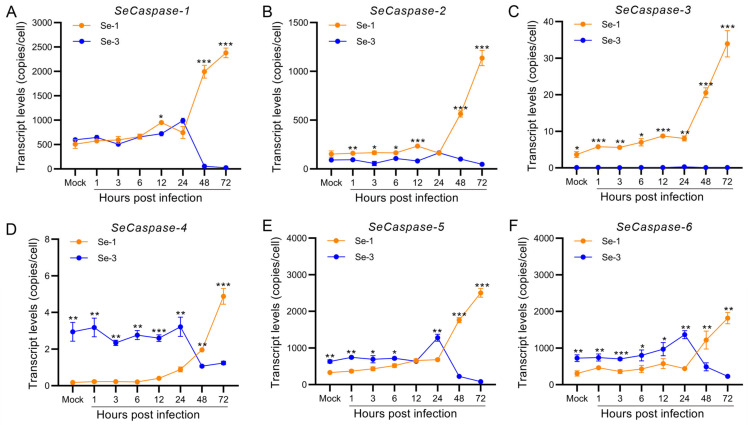
Transcription analysis of the six *caspase* genes in two different *S. exigua* cell lines during AcMNPV infection. Se-1 and Se-3 cells were infected with AcMNPV at an MOI of 5. At 1, 3, 6, 12, 24, 48 and 72 h p.i., the transcript levels of the six caspase genes of *S. exigua*, *SeCaspases-1* (**A**), *SeCaspase-2* (**B**), *SeCaspases-3* (**C**), *SeCaspase-4* (**D**), *SeCaspases-5* (**E**), and *SeCaspase-6* (**F**), in the two infected cells were detected by RT-qPCR using gene-specific primers. Mock cells were the negative control, with no virus infection. Each test was performed three times. The error bars indicate the standard error of the mean (SEM) from the means of triplicates. The asterisk represents a significant difference between the two cell lines at the same time point. *, *p* < 0.05; **, *p* < 0.001; ***, *p* < 0.0001.

To analyse the apoptosis differences in the cell gene expression of the two infected cell lines, we measured the transcriptional levels of the six core apoptosis genes, *S. exigua* Caspase-1 to -6 (SeCaspase-1 to -6), by reverse transcription quantitative PCR (RT-qPCR). The results showed that the transcription levels of these six genes were different between the Se-1 and Se-3 cell lines (Figure 2). Among them, the transcription levels of SeCaspase-1 and SeCaspase-2 in Se-1 cells were low and similar to those in Se-3 cells at 1 h to 24 h p.i. However, they significantly increased in Se-1 cells but decreased in Se-3 cells at 48 h to 72 h p.i. (Figure 2A,B). The transcription level of SeCaspase-3 increased slowly at 1 h to 24 h p.i. and then significantly increased at 48 h to 72 h p.i. in Se-1 cells, whereas its transcription level was very low during viral infection in Se-3 cells (Figure 2C). The transcription levels of SeCaspase-4, -5 and -6 in Se-1 cells were lower than those in Se-3 cells at 1 h to 24 h p.i. However, they increased in Se-1 cells and were significantly higher than those in Se-3 cells at 48 h to 72 h p.i. (Figure 2D–F). These results indicated that SeCaspase-1, -2 and -3 were all activated in Se-1 cells but inhibited in Se-3 cells during AcMNPV infection. SeCaspase-4, -5 and -6 were all also activated in Se-1 cells, and even though they were slightly activated in Se-3 cells at the early stages of infection, they were significantly inhibited at the late stages of infection.

### 2.4. Transcription Analysis of the Antiapoptotic Genes in Two Different S. exigua Cell Lines during AcMNPV Infection

**Figure 3:**
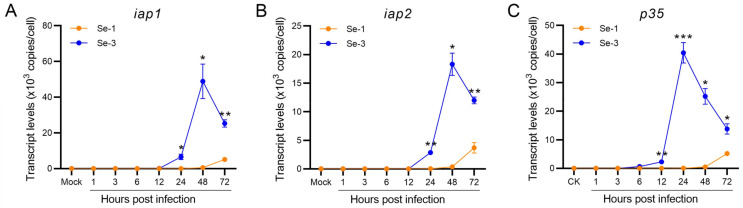
Transcription analysis of the antiapoptotic genes in two different *S. exigua* cell lines during AcMNPV infection. Se-1 and Se-3 cells were infected with AcMNPV at an MOI of 5. At 1, 3, 6, 12, 24, 48 and 72 h p.i., the transcript levels of *iap1* (**A**), *iap2* (**B**) and *p35* (**C**) in the two infected cells were measured by RT-qPCR using their gene-specific primers. Mock cells were the negative control, with no virus infection. Each test was performed three times. The error bars indicate the standard error of the mean (SEM) from the means of results for triplicates. The asterisk represents a significant difference between the two cell lines at the same time point. *, *p* < 0.05; **, *p* < 0.001; ***, *p* < 0.0001.

To investigate whether the baculovirus antiapoptotic proteins affected the apoptosis differences in the two infected cell lines, we also determined the transcription levels of the three antiapoptotic protein genes of AcMNPV (iap1, iap2 and p35) by RT-qPCR. The results showed that the transcription levels of these three antiapoptotic genes were very different between the Se-1 and Se-3 cell lines (Figure 3). The transcription levels of iap1 and iap2 in Se-1 cells were very low at 1 h to 48 h p.i. and then increased at 48 h to 72 h p.i., whereas they were also very low at 1 h to 12 h p.i. in Se-3 cells, but they significantly increased at 24 h to 48 h p.i. (Figure 3A,B). Even though the transcription levels of iap1 and iap2 decreased gradually at 72 h p.i., they also maintained a high level (Figure 3A,B). The transcription level of p35 in the two cell lines was similar to that of iap1 and iap2, but it increased and peaked earlier than that of iap1 and iap2 (Figure 3A–C). These results suggested that p35 played a major role in the apoptosis inhibition of Se-3 cells, and the inhibition of p35 expression might induce apoptosis of Se-1 cells during AcMNPV infection.

### 2.5. Expression of P35 Protein in Two Different S. exigua Cell Lines Infected with Recombinant AcMNPV

**Figure 4:**
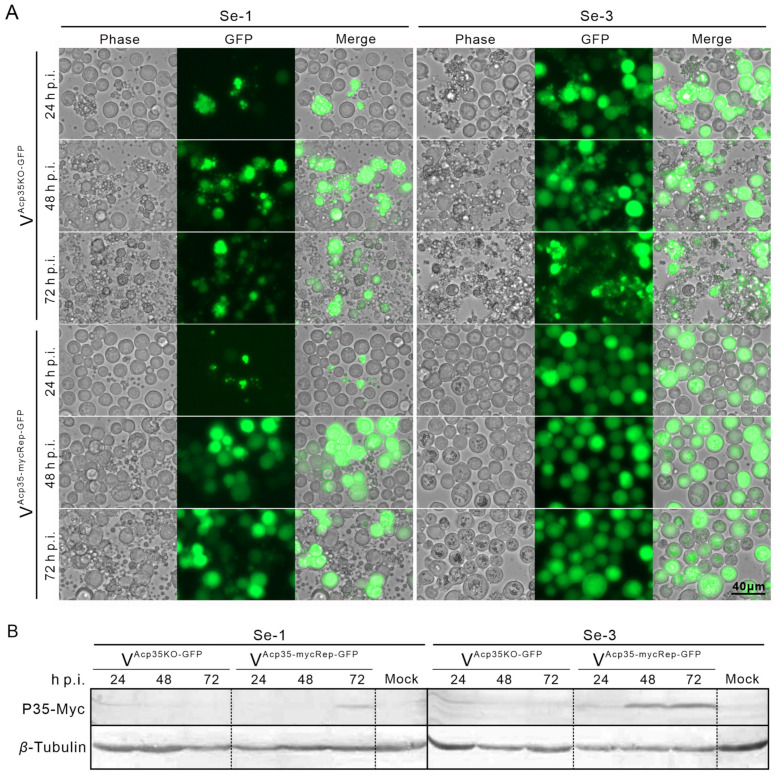
Apoptosis analysis and expression of P35 protein in two different *S. exigua* cell lines infected with recombinant AcMNPV. (**A**) Apoptosis analysis of the two cell lines. Se-1 and Se-3 cells were infected with a *p35* knockout AcMNPV (V^Acp35KO-GFP^) at an MOI of 0.5 and a myc-tagged *p35* repair AcMNPV (V^Acp35-mycRep-GFP^) at an MOI of 1. At 24, 48 and 72 h p.i., apoptosis and virus infections were observed under a fluorescence microscope. (**B**) Western blot analysis of the expression of the P35 protein in the two cell lines. A parallel group of infected Se-1 and Se-3 cells were also lysed at 24, 48 and 72 h p.i., and the expression of P35 proteins was detected by anti-Myc and anti-*β*-tubulin monoclonal antibodies. Mock cells were the negative control, with no virus infection.

To verify whether the expression of p35 was associated with the apoptosis differences in the two cell lines, we infected these cells with the recombinant viruses, the p35 knockout AcMNPV (V^Acp35KO-GFP^) at an MOI of 0.5 and the myc-tagged p35 repair AcMNPV (V^Acp35-mycRep-GFP^) at an MOI of 1. We then observed the apoptosis phenomenon. We found that, after infection with V^Acp35KO-GFP^, both Se-1 and Se-3 cells underwent severe apoptosis with the production of apoptotic bodies (Figure 4A). In comparison, after infection with V^Acp35-mycRep-GFP^, only Se-1 cells still underwent severe apoptosis, whereas Se-3 cells did not undergo apoptosis (Figure 4A). At the same time, after infection with V^Acp35KO-GFP^, no viral OBs were produced in Se-1 cells, and only a few OBs were produced in Se-3 cells. However, after infection with V^Acp35-mycRep-GFP^, no OBs were produced in Se-1 cells, but OBs were produced normally in Se-3 cells (Figure 4A). In addition, a Western blot assay was performed to detect P35 protein expression in the two cell lines. The results showed that after infection with V^Acp35KO-GFP^, no P35 was expressed in either Se-1 or Se-3 cells (Figure 4B). When infected with V^Acp35-mycRep-GFP^, P35 was expressed only at 72 h p.i. in Se-1 cells and at 24 to 72 h p.i. in Se-3 cells (Figure 4B). These results indicated that the different expression of P35 is an important reason for the apoptosis differences in Se-1 and Se-3 cell lines induced by AcMNPV infection.

### 2.6. Transcription Analysis of SeCaspases in Se-3 Cell Lines during Recombinant V^Acp35KO-GFP^ Infection

**Figure 5:**
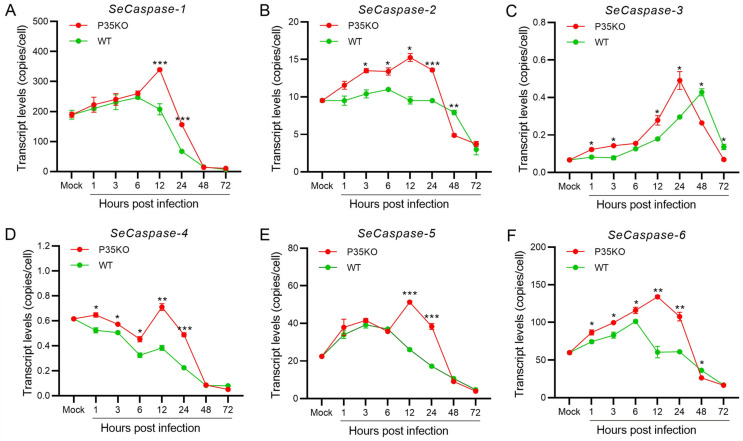
Transcription analysis of the six caspase genes in Se-3 cell lines during recombinant virus V^AcP35KO-GFP^ infection. Se-3 cells were infected with WT-AcMNPV and V^Acp35KO-GFP^ at an MOI of 0.5. At 1, 3, 6, 12, 24, 48 and 72 h p.i., the transcript levels of *SeCaspases-1* (**A**), *SeCaspase-2* (**B**), *SeCaspases-3* (**C**), *SeCaspase-4* (**D**), *SeCaspases-5* (**E**), and *SeCaspase-6* (**F**) were measured by RT-qPCR. Mock cells were the negative control, with no virus infection. Each test was performed three times. The error bars indicate the standard error of the mean (SEM) from the means of triplicates. The asterisk represents a significant difference between the two cell lines at the same time point. *, *p* < 0.05; **, *p* < 0.001; ***, *p* < 0.0001.

To explore the relationship between the expression of p35 and SeCaspase-1 to -6, we infected Se-3 cells with WT-AcMNPV and the recombinant virus V^Acp35KO-GFP^ at an MOI of 0.5. Then, the transcriptional levels of the six SeCaspases were measured by RT-qPCR. We found that, after infection with the recombinant virus V^Acp35KO-GFP^, the transcription levels of the six caspase genes were all significantly increased and higher than those after infection with WT-AcMNPV (Figure 5). Among them, after infection with V^Acp35KO-GFP^, the transcription levels of SeCaspase-1, -2, -4, -5 and -6 were all significantly increased at 1 to 12 h p.i. and decreased at 24 to 72 h p.i. (Figure 5A,B,D–F). The transcription level of SeCaspase-3 was significantly increased at 1 to 24 h p.i. and decreased at 48 to 72 h p.i. (Figure 5C). It is worth noting that, after infection with V^Acp35KO-GFP^, the transcription levels of SeCaspase-1 and -5 at 12 to 24 h p.i. were all significantly higher than those after infection with WT-AcMNPV (Figure 5A,E). The transcription levels of SeCaspase-2, -3, -4 and -6 at 1 to 24 h p.i. were all significantly higher than those after infection with WT-AcMNPV (Figure 5B–D,F). These results showed that SeCaspase-1 to -6 in Se-3 cells were all activated in the absence of p35 during recombinant virus infection. Combined with the results of 2.3 and 2.5, we suggested that the normal expression of P35 was crucial for inhibiting SeCaspases and suppressing the apoptosis induced by AcMNPV infection.

### 2.7. Expression Change Analysis of the Genes of the Two Different S. exigua Cell Lines during AcMNPV Infection

To comprehensively explore the reasons for the apoptosis differences between Se-1 and Se-3 cells, we were also interested in exploring what affected the expression of p35 in Se-1 cells. Therefore, we performed transcriptome analysis of the Se-1 and Se-3 cell lines infected with AcMNPV. Based on the results, the two cell lines showed apoptosis differences during AcMNPV infection (Figure 1), and the expression level of p35 in Se-3 cells was significantly increased compared with that in Se-1 cells at 12 h p.i. (Figure 3C). Therefore, we only showed the transcriptome analysis of the two cell lines at 1 and 12 h p.i. We focused on the DEGs from apoptosis-associated pathways, such as apoptosis, the P53 pathway, the JAK-STAT signalling pathway, the PI3K-Akt pathway and the NIK-NF-kB pathway. Compared to Se-3 cells, the genes upregulated and downregulated in Se-1 cells are listed in Table 1. These genes were likely to play an important role in the differences in apoptosis between the two cell lines. Among them, the Stat involved in the JAK/STAT pathway has been suggested to regulate the expression of p35 and affect virus proliferation [16,37]. In our results, Stat (SeL31G020844) in Se-1 cells was expressed at lower levels than that in Se-3 cells. Therefore, we speculated that Stat (SeL31G020844) may also be involved in regulating the expression of p35, and perhaps any other DEGs can also regulate p35 expression.

## 3. Discussion

Baculovirus infection induces the apoptosis of host cells, which can reduce its production and infection. Conversely, baculoviruses can also regulate the caspase gene to inhibit the apoptosis induced by their infection [7]. Prior studies showed that the core caspase genes of lepidopterans and the major antiapoptotic protein P35 of AcMNPV were involved in apoptosis induced and inhibited by the virus [23,38,39,40]. In our study, we found that AcMNPV infection induced different apoptotic phenomena in two different *S. exigua* cell lines. This laid a good foundation for a deeper exploration of the mechanisms of apoptosis regulated by baculoviruses. Therefore, we further analysed the different apoptosis responses, including the different expression of *SeCaspase-1* to *-6* and the antiapoptotic gene AcMNPV in the two cell lines of *S. exigua*. We also analysed the relationship between the expression of *SeCaspase-1* to *-6* and *p35.* We found that the different expression of *p35* was probably an important reason for the different apoptosis in the two cell lines induced by AcMNPV infection.

Further analysis of the apoptosis process showed that Se-1 cells underwent apoptosis during the entire AcMNPV infection (Figure 1B, Se-1 panel). Even though Se-3 cells underwent very slight apoptosis at the early stage of infection (1–6 h p.i.), the apoptosis rate suggested that the apoptosis of Se-3 cells was slighter than that of Se-1 cells (Figure 1C). However, at the late stage of infection (12–72 h p.i.), the apoptosis in Se-1 cells was more severe, and a large number of cells formed apoptotic bodies, while the slight apoptosis in Se-3 cells was suppressed (Figure 1B). Combined with the results that virus production in Se-1 cells was reduced but normal in Se-3 cells, we suggested that AcMNPV infection induced apoptosis in both Se-1 and Se-3 cell lines. Additionally, the apoptosis of Se-3 cells was inhibited by the virus without affecting virus production, but the apoptosis of Se-1 cells was not inhibited, which in turn affected virus production.

Previous studies have shown that lepidopteran caspases are involved in baculovirus-induced apoptosis [38,39,40]. Based on the transcriptome of *S. exigua* and the analysis of phylogeny and sequence with caspase genes of *Drosophila* and other lepidopteran insects, we identified six caspase genes in *S. exigua*, *SeCaspase-1* to *-6*. Similarly, the analysis of transcriptional levels showed that *SeCaspase-1* to *-6* were all activated in Se-1 cells, whereas they were all inhibited in Se-3 cells (Figure 2). This result suggested that the apoptosis in Se-1 cells induced by AcMNPV infection was probably due to activation of the six *SeCaspase* genes, and the apoptosis inhibition in Se-3 cells was probably due to inhibition of the six *SeCaspase* genes. However, which *SeCaspase* played the major role in AcMNPV infection-induced apoptosis deserves further consideration. To date, regarding the six *caspase* genes of lepidopterans (*LepCaspase-1* to *-6*), only the functions of three have been verified [19]. *LepCaspase-1* is an effector caspase that is involved in the classical apoptosis pathway and participates in baculovirus-induced apoptosis [21,39,41]. *LepCaspase-5* and *LepCaspase-6* are initiator caspases [22,42,43]. However, whether they are both involved in the classical apoptosis pathway or participate in baculovirus-induced apoptosis is still unknown. *LepCaspase-2*, *LepCaspase-3* and *LepCaspase-4* have been identified and sequenced, but their functions are still unclear [19]. Interestingly, our recent study found that the apoptotic morphology induced by *SeCaspase-4* overexpression in Se-3 cells was very different from that induced by *SeCaspase-1* and *SeCaspase-5*, suggesting that SeCaspase-4 might not be involved in the classical apoptosis pathway. Based on the finding that *SeCaspase-1* to -*6* were all activated in Se-1 cells and inhibited in Se-3 cells (Figure 2), it was likely that they might work together and that there may be different apoptotic pathways involved in AcMNPV-induced apoptosis in the two cell lines.

Baculoviruses typically inhibit apoptosis by encoding antiapoptotic proteins, and they can inhibit caspase proteins by directly interacting with them to prevent apoptosis [23]. In AcMNPV, perhaps only P35 plays a major role in inhibiting apoptosis during its infection because IAP1 and IAP2 did not inhibit apoptosis in some AcMNPV-infected cell lines [35,36]. In this study, all three antiapoptotic genes, *iap1*, *iap2* and *p35*, were expressed normally in Se-3 cells but were inhibited in Se-1 cells. However, *p35* was highly expressed and accumulated earlier than *iap1* and *iap2* (Figure 3). These results indicated that *p35* probably played a more prominent role than *iap1* or *iap2* in inhibiting the apoptosis of Se-3 cells. Therefore, we asserted that the differential expression of the *p35* gene was an important reason for the apoptosis differences in Se-1 and Se-3 cell lines induced by AcMNPV infection.

Deletion of *p35* induced severe apoptosis in SF21 cells when infected with recombinant AcMNPV, but repair of *p35* could save SF21 cells from apoptosis [35,36]. The P35 protein was also beneficial for AcMNPV inhibition of apoptosis and promoted the production of progeny viruses [44,45]. However, in this study, the infection of V^Acp35KO-GFP^ (*p35* knockout AcMNPV) induced severe apoptosis both in Se-1 and Se-3 cell lines, but the V^Acp35-mycRep-GFP^ (*p35* repair AcMNPV) infection only induced apoptosis in Se-1 cells (Figure 4A). The expression of P35 protein was inhibited in Se-1 cells but normal in Se-3 cells (Figure 4B). These results demonstrated that the different expression of P35 was relevant to the apoptosis differences in Se-1 and Se-3 cells. Otherwise, based on earlier studies showing that P35 can inhibit the activation of effector caspases by interacting with them [46,47,48] and our findings that the expression trends of *p35* and the six *SeCaspase* genes were completely opposite in the two cell lines (Figure 2 and Figure 3), we proposed that the expression of *SeCaspases* might be associated with the expression of *p35*. Therefore, we infected Se-3 cells with the recombinant virus V^Acp35KO-GFP^ and found that the expression of the six *SeCaspases* was not affected in the apoptosis-inhibited Se-3 cells (Figure 2 and Figure 5). However, whether there is a direct interaction between P35 and the six SeCaspase proteins needs to be further studied.

Given the different expression of *p35* between Se-1 and Se-3 cell lines during AcMNPV infection, an interesting question was raised: Who can regulate the expression of *p35* in Se-1 cells? Transcriptome analysis of the two cell lines infected with AcMNPV showed that many genes associated with apoptosis were differentially expressed in the two cell lines (Table 1). Among these genes, *Stat* has been reported to regulate baculovirus-induced apoptosis, and silencing *Stat* reduced the expression of *p35*, which cannot inhibit the cell apoptosis induced by viral infection [16,37]. Interestingly, in our findings, *Stat* (SeL31G020844) expression was lower in Se-1 cells than in Se-3 cells (Table 1, 1 h p.i.), which suggested that the low expression of *Stat* (SeL31G020844) might reduce the expression of *p35* at the early stage of infection and further affect the significant accumulation of *p35*. However, the function and mechanism of *Stat* (SeL31G020844) in regulating *p35* expression need to be further tested. Otherwise, further research is required to investigate whether there are any other upstream regulators of *p35* in the DEGs and understand their mechanisms.

In conclusion, we determined that AcMNPV infection induced different apoptotic responses in two different *S. exigua* cell lines. The expression of *SeCaspase-1* to *-6* and *p35* in Se-1 and Se-3 cell lines was analysed because of the apoptosis differences induced by AcMNPV infection. The relationship between *SeCaspase-1* to *-6* and *p35* was detected. These results suggested that the expression of P35 played a key role in the apoptosis differences induced by AcMNPV infection between the two cell lines. The analysis of DEGs in the two cell lines indicated that some genes from apoptosis-associated pathways might be involved in regulating the expression of *p35*. We will continue to identify the upstream regulators of *p35* and study their mechanisms in the future. This will shed light on the molecular mechanism of baculovirus-induced apoptosis and be beneficial for improving the control effects of baculovirus insecticides.

## 4. Materials and Methods

### 4.1. Cell Lines and Viruses

The *S. exigua* cell lines Se-1 and Se-3 were established by our laboratory [49], and the *S. frugiperda* cell line Sf9 was provided by Dr. Granados of Cornell University, Ithaca, NY, USA. These cell lines were cultured at 27 °C with TNM-FH medium (Procell Life Sciences & Technology Co., Ltd., Wuhan, China) supplemented with 10% foetal bovine serum (FBS) (Biological Industries, Kibbutz Beit-Haemek, Israel). Wild-type AcMNPV was provided by Dr. Granados of Cornell University, Ithaca, NY, USA, and amplified and stored in our laboratory. The recombinant viruses V^Acp35KO-GFP^ and V^Acp35mycRep-GFP^ were provided by Dr. Guo from Northwest A&F University, Yangling, China. The p35 knockout bacmid (V^Acp35KO-GFP^) was generated using ET homologous recombination. Briefly, a 1016 bp fragment containing the chloramphenicol acetyltransferase (CAT)-expressing cassette was PCR amplified using the pKD3 plasmid as a template and then inserted into pIE-HA-MCS [50] by digestion with BamHI and PstI to generate the pIE-CAT vector. Then, the homologous arms of 225 bp and 397 bp were amplified, separately digested with SacI/BamHI and PstI/HindIII and inserted into pIE-CAT to generate pIE-35U-CAT-35D. Finally, the cassette containing the CAT fragment and the homologous arms was isolated from pIE-35U-CAT-35D by digestion with SacI and HindIII and then transformed into BW25113 competent cells harbouring wild-type (WT) AcMNPV bacmid (bMON14272) and pKD46 plasmid to replace the complete ORF and 5′-upstream transcription start sequence (27 bp) of p35 for screening the p35 knockout bacmid. To construct the p35 repaired bacmid, a fragment containing the ORF and the native promoter of p35 was amplified using the primers p35NPSKF (5′-AATGAGCTCGGTACCTACACGCGCGTCGTAACGTG-3′) and p35ER (5′-ATAGAATTCTTATTTAATTGTGTTTAATATTACAT-3′) and inserted into pIE-HA-MCS by digestion with SacI and EcoRI to generate the plasmid pIE-P35-p35. Then, the fragment containing the p35 promoter, the ORF of p35, and the poly(A) sequence of the AcMNPV gp64 gene was isolated by digestion with KpnI and HindIII and inserted into Ppro-GFP-MCSpFB [51]. After transformation into DH10B competent cells containing the helper plasmid pMON7124 and the p35 knockout bacmid (AcMNPV^p35KO^), the repaired bacmid (AcMNPV^p35Rep^) was generated by site-specific transposition as described previously [52]. The two bacmids AcMNPV^p35KO^ and AcMNPV^p35Rep^ were transfected into Sf9 cells to generate the recombinant virus V^Acp35KO-GFP^ and V^Acp35mycRep-GFP^. These viruses were amplified and titrated in Se-3 cells.

### 4.2. Virus Infection and Productions Determination

Se-1 and Se-3 cells were seeded at a density of 1 × 10^6^ cells per well in 6-well plates for 24 h. The cells were then inoculated with WT-AcMNPV at a multiplicity of infection (MOI) of 5 for 1 h. After removing the viral inoculum, the cells were washed once with TNMFH (10% FBS) medium and then incubated at 27 °C. At different time points (24, 48 and 72 h p.i.), the two infected cell lines were observed and photographed under a microscope (EVOS FL, Thermo Fisher Scientific, Waltham, MA, USA) to analyse the apoptosis phenomenon and formation of apoptotic bodies. Then, the precipitated infected cells containing OBs and the cell culture supernatants containing BVs were collected separately. The BV titers were determined by half-tissue culture infection dose (TCID_50_) assays. The OB production was calculated as described in reference [53].

### 4.3. Apoptosis Process and Rate Analysis

Se-1 and Se-3 cells were seeded at a density of 2 × 10^5^ cells per well in 24-well plates for 24 h and then inoculated with WT-AcMNPV at an MOI of 5 for 1 h. After removing the viral inoculum, the cells were washed once with TNMFH (10% FBS) medium and then incubated. At different time points (1, 3, 6, 12, 24, 48 and 72 h) p.i., the infected cells were examined with an Annexin V-FITC/PI Cell Apoptosis Detection Kit (TransGen) and then observed and photographed under a fluorescence microscope (EVOS FL, Thermo Fisher Scientific, Waltham, MA, USA). The apoptotic process was divided into early apoptosis and late apoptosis. Cells in which only the membrane is stained green with FITC are in early apoptosis, while cells in which the nuclei are also stained red with PI are in late apoptosis. The apoptosis rates were calculated using randomly taken photos. The percentage of the total number of green and red cells to the total number of seeded cells was used as the apoptosis rate.

### 4.4. Expression Analysis of Cell and Virus Genes by Reverse Transcription Quantitative PCR

Se-1 and Se-3 cells were seeded in 24-well plates and infected with WT-AcMNPV as described above in Results 2.3 and 2.4. And Se-3 cells were seeded at a density of 6.5 × 10^5^ cells per well in 12-well plates for 24 h and then inoculated with WT-AcMNPV and the recombinant virus V^Acp35KO-GFP^ at an MOI of 0.5 in Result 2.6. At different time points (1, 3, 6, 12, 24, 48 and 72 h) p.i., the total RNA of the infected cells was extracted with TRIzol reagent (Invitrogen, Carlsbad, CA, USA), and 1 μg RNA was reverse transcribed using the PrimerScript RT Reagent Kit with gDNA Eraser (Takara, Bio Inc., Dalian, China). The quantifications of the six genes (*SeCaspase-1* to *-6*) and three AcMNPV antiapoptotic genes (*iap1*, *iap2* and *p35*) were conducted by reverse transcription quantitative PCR (RT-qPCR) using ChamQ Universal SYBR qPCR Master Mix (Nanjing Vazyme Medical Technology Co., Ltd., Nanjing, China). Each PCR mixture contained 10 μL of 2× ChamQ universal SYBR qPCR master mix, 0.4 μL of each primer (10 μM) and 2 μL of cDNA (equal to 100 ng of total RNA). The reaction conditions were 95 °C for 2 min, followed by 40 cycles at 95 °C for 15 s and 60 °C for 30 s. Each test was performed three times. Sample analysis was performed on Analytik Jena’s qPCR soft 4.0 system (Analytik Jena GmbH, Jena, Germany). The specific primers for these nine genes are listed in Table 2. The different DNA fragments of these nine genes were amplified with these specific primers and cloned into the pMD18-T vector (Takara, Bio Inc., Dalian, China) to construct the plasmids (Table 1), which were used to generate the standard curve. The expression levels of these nine genes were calculated in accordance with the absolute quantity standard curve.

### 4.5. Western Blot Analysis

Se-1 and Se-3 cells were seeded at a density of 5 × 10^5^ cells per well in 12-well plates for 24 h and then infected with the recombinant viruses V^Acp35KO-GFP^ and V^Acp35-mycRep-GFP^ at MOIs of 0.5 and 1. However, when only infected with V^Acp35KO-GFP^ at an MOI of 0.5 and V^Acp35-mycRep-GFP^ at an MOI of 1, the apoptosis phenomenon of Se-1 cells was similar in that the apoptosis differences and the expression of P35 protein in the two cell lines could be effectively compared. At different times (24, 48 and 72 h p.i.), one group of the two infected cells was observed and photographed to analyse the processes of virus infection and cell apoptosis. The other group of infected cells was collected and lysed in Triton X-100 lysis buffer (150 mM NaCl, 0.1% Triton X-100, 50 mM Tris, pH 8.0) containing the complete protease inhibitor cocktail (Roche Applied Science), followed by analysis using 10% SDS-polyacrylamide gels under reducing conditions, and transferred to a 0.20 μm polyvinylidene difluoride (PVDF) membrane (Millipore). The P35-Myc (approximately 35 kDa) fusion proteins on the membrane were incubated with anti-Myc mouse monoclonal antibodies (ABclonal) (1:1000), and *β*-tubulin (50 kDa) proteins were detected with anti-*β*-tubulin (Abbkine) (1:2000) and then detected with alkaline phosphatase-conjugated goat anti-mouse IgG (ABclonal) (1:5000). Additionally, the immunoreactive proteins were visualised by using the substrates 5-bromo-4-chloro-3-indolyphosphate (BCIP, Promega) and nitro-blue-tetrazolium (NBT, Promega).

### 4.6. Analysis of the Differentially Expressed Genes from Apoptosis-associated Pathways

Se-1 and Se-3 cells were seeded at 1 × 10^6^ cells per well in 6-well plates and infected with AcMNPV at an MOI of 5. At different time points (1, 3, 6, 12, 24, 48 and 72 h) p.i., the total RNA of the infected cells was extracted with TRlzol reagent (Invitrogen, Carlsbad, CA, USA), and RNA degradation and contamination were monitored on 1% agarose gels. A 3 μg quantity of RNA per sample was used as input material for preparing the cDNA library, which was sequenced by Beijing Genomics Institution (BGI Genomics Co., Ltd., Shenzhen, China) using HiSeq 2500. The final unigenes were obtained by raw read filtering, clean read merging and splicing twice. The *S. exigua* reference genome was downloaded from GenBank (GCA_022315195.1) [54]. Clean data were mapped to the reference genome using HISAT2 (version 2.2.1) [55]. Only data uniquely mapping to reference sequence reads were retained for subsequent analyses. Gene function was annotated based on the Gene Ontology database (GO) and the Kyoto Encyclopedia of Genes and Genomes (KEGG). Gene expression levels were calculated using the TPM (transcripts per million reads) method. The differentially expressed genes (DEGs) from Se-1 cells compared to those of Se-3 cells were detected by DEGseq [56] with |log2 (fold change)| > 1 and *p*-value < 0.005. Then, the DEGs were subjected to KEGG (http://www.genome.jp/kegg/ (accessed on 20 February 2023)) enrichment analyses using an online website KOBAS3.0 (http://kobas.cbi.pku.edu.cn/ (accessed on 20 February 2023)).

### 4.7. Statistical Analysis

All data were analysed using SPSS 21.0 software (IBM, Armonk, NY, USA). The results were expressed as the standard error of the mean (SEM) from the means of triplicates. We used the Student’s *t*-test to examine the statistically significant differences (*, *p* < 0.05; **, *p* < 0.001; ***, *p* < 0.0001) between the mean values. We used analysis of variance (ANOVA) for the transcript levels of *SeCaspase-1* to *-6* in the Se-3 cell line infected with the recombinant virus V^Acp35KO-GFP^. We used Tukey (honestly significant difference, HSD) post hoc tests to determine whether the transcript levels of the six *SeCaspases* at the different time points during viral infection differed (*p* < 0.05). The figures of data were created using GraphPad Prism 7 (GraphPad, San Diego, CA, USA).

## Figures and Tables

**Table 1:** Expression change analysis of the genes in some apoptosis-associated pathways between the two different *S. exigua* cell lines during AcMNPV infection.

Gene Name	KEGG ID (Name)	Gene ID	Log2 (Fold Change)		
			Mock	1 h p.i.	12 h p.i.
**Apoptosis**					
*Aif*	K04727(AIFM1)	SeL15G011396	−1.8	nc	−3.4
*dTspo*	K05770(TSPO)	SeL24G017306	nc	nc	1
*Bicaudal*	K01527(EGD1)	SeL24G017329	nc	nc	1.2
*Lamin*	K07611(LMNB)	SeL02G001429	nc	nc	1.2
*Buffy*	K20017(BUFFY)	SeL04G003073	2.7	2.0	3.5
*Drp1*	K17065(DNM1L)	SeL13G009814	nc	nc	1.4
*Htra2*	K08669(HTRA2)	SeL24G017609	nc	nc	1.1
*Apaf-1*	K02084(APAF1)	SeL09G006996	nc	−1.5	nc
**P53 pathway**					
*Mdm2*	K06643(MDM2)	SeL09G006582	nc	1	3
*Pigs*	K10133(TP53I3)/K10134(EI24)	SeL07G005232	nc	nc	1
*Siah*	K04506(SIAH1)	SeL06G005033	nc	−1.5	−1.2
		SeL03G002261	1.1	nc	1.3
**JAK-STAT signalling pathway**					
*Stat1-6*	K11220(STAT1)/K11221(STAT2)/K04692(STAT3)/K11222(STAT4)/K11223(STAT5A, B)/K11225(STAT6)	SeL31G020844	nc	−1.1	nc
*Pias1-2*	K04706 (PIAS1)/K16063(PIAS2)	SeL27G018744	nc	nc	−1.6
		SeL08G006116	nc	nc	1.1
*Cbp/p300*	K04498(EP300)	SeL31G020863	−1.6	−1.6	−1.1
*Cis*	K04701(CISH)	SeL17G012537	nc	−1.2	3.4
*Socs*	K04694(SOCS1)/K04695(SOCS2)/K04696(SOCS3)/K04697(SOCS4)/K04698(SOCS5)/K04699(SOCS6_7)	SeL11G008507	−1.3	nc	1.9
		SeL29G019826	1.1	1	2.4
		SeL01G000204	nc	−1.1	nc
**PI3K-Akt pathway**					
*Pi3k*	K00929(PIK3CA_B_D)/K02649(PIK3R1_2_3)	SeL05G003720	−1.5	−1.1	−1.6
		SeL07G005570	nc	−1.1	nc
		SeL06G004934	nc	nc	1.1
*Pten*	K01110(PTEN)	SeL18G013590	nc	nc	1
*Magi*	K05629(AIP1)/K05631(AIP3)	SeL26G018583	nc	nc	1
*Pdk1*	K06276(PDPK1)	SeL20G014856	nc	nc	1
*Pp2a*	K04382(PPP2C)/K03456(PPP2R1)/K04354(PPP2R2)/K11583(PPP2R3)/K11584(PPP2R5)	SeL19G014057	nc	−1.2	−1.1
		SeL24G017678	1.3	1.3	1.9
		SeL03G002068	nc	nc	2.5
*Cdc37*	K09554(CD37)	SeL13G009955	−1.5	−1.1	−1.2
*Phlpp*	K16340(PHLPP)	SeL04G003119	nc	nc	−4.5
*Creb*	K05870(CREB1)/K04450(ATF2)/K04374(ATF4)/K09048(CREB3)/K09047(CREB5)/K09049(ATF6B)	SeL13G009595	nc	nc	3
		SeL17G012601	−1.1	−1.3	nc
**NIK-NF-kB pathway**					
*Ikk*	K04467(IKBKA)	SeL18G013302	nc	nc	−3.2
	K07209(IKBKB)	SeL04G003156	−1.3	−1.5	nc

**Note:** Differentially expressed genes (DEGs) are only reported if statistically significant probe intensity differences exist (*p* < 0.05 via unpaired two-tailed *t*-test). Mock cells were the negative control, with no virus infection. The positive and negative numbers represent the upregulation and downregulation of gene expression in Se-1 cells compared to Se-3 cells, respectively. nc, no change.

**Table 2:** RT-qPCR primers.

Primer Name	Sequences (5′to 3′)	Plasmids
SeCaspase-1qF	gcgaggatgccagtggataga	pMD18-T-SeCaspase-1
SeCaspase-1qR	ccttgaggactttggataggttgt	
SeCaspase-2qF	ccgagacagacggctggtat	pMD18-T-SeCaspase-2
SeCaspase-2qR	cacggtcacggtttctccac	
SeCaspase-3qF	aatgccttgtacgacatgagc	pMD18-T-SeCaspase-3
SeCaspase-3qR	ggttcttgttgatggctgctt	
SeCaspase-4qF	atctgcctcggcttggga	pMD18-T-SeCaspase-4
SeCaspase-4qR	cgccacgcagccatagtc	
SeCaspase-5qF	ttgcccatctggtagacgcttta	pMD18-T-SeCaspase-5
SeCaspase-5qR	ctgctgttcttgttcgggttgta	
SeCaspase-6qF	tttcaacgagtttccaacgatg	pMD18-T-SeCaspase-6
SeCaspase-6qR	tgcattgacattcagtcccagt	
IAP1qF	gtgcgagtattgcgaagcag	pMD18-T-IAP1
IAP1qR	acacacacttgggtttgcct	
IAP2qF	taggctgtcgcacgattttg	pMD18-T-IAP2
IAP2qR	tgtcaacgaccacggattgt	
P35qF	tcgacgtgtcccagacgatt	pMD18-T-P35
P35qR	cttgcgcgtaacgcttcgta	

## Data Availability

Data are contained within the article.
